# Production of Inhalable Ultra-Small Particles for Delivery of Anti-Inflammation Medicine via a Table-Top Microdevice

**DOI:** 10.3390/mi13091382

**Published:** 2022-08-25

**Authors:** Matthew J. Owen, Umit Celik, Subash K. Chaudhary, Jasper H. N. Yik, John S. Patton, Mei-chang Kuo, Dominik R. Haudenschild, Gang-yu Liu

**Affiliations:** 1 Department of Chemistry, University of California, Davis, CA 95616, USA; 2 Tesio Pharmaceuticals, Inc., Davis, CA 95616, USA; 3 Department of Orthopedic Surgery, School of Medicine, University of California Davis, Sacramento, CA 95817, USA

**Keywords:** Flavopiridol, inhalable particles, pulmonary delivery, inflammation, drug release, COVID

## Abstract

A table-top microdevice was introduced in this work to produce ultrasmall particles for drug delivery via inhalation. The design and operation are similar to that of spray-drying equipment used in industry, but the device itself is much smaller and more portable in size, simpler to operate and more economical. More importantly, the device enables more accurate control over particle size. Using Flavopiridol, an anti-inflammation medication, formulations have been developed to produce inhalable particles for pulmonary delivery. A solution containing the desired components forms droplets by passing through an array of micro-apertures that vibrate via a piezo-electrical driver. High-purity nitrogen gas was introduced and flew through the designed path, which included the funnel collection and cyclone chamber, and finally was pumped away. The gas carried and dried the micronized liquid droplets along the pathway, leading to the precipitation of dry solid microparticles. The formation of the cyclone was essential to assure the sufficient travel path length of the liquid droplets to allow drying. Synthesis parameters were optimized to produce microparticles, whose morphology, size, physio-chemical properties, and release profiles met the criteria for inhalation. Bioactivity assays have revealed a high degree of anti-inflammation. The above-mentioned approach enabled the production of inhalable particles in research laboratories in general, using the simple table-top microdevice. The microparticles enable the inhalable delivery of anti-inflammation medicine to the lungs, thus providing treatment for diseases such as pulmonary fibrosis and COVID-19.

## 1. Introduction

Inflammation in the lungs is a principal cause of diseases, including lung fibrosis [1,2], lung cancer [3,4], and (more recently) lung failure due to COVID-19, and even of death [5,6]. Therefore, anti-inflammation represents an important target of medical interventions [7,8]. Prior work done by us and others have identified a potent anti-inflammation therapeutic, Flavopiridol [9,10,11,12,13,14], a well-known small molecule inhibitor for cyclin dependent kinase 9 inhibitor (CDK9). It was originally designated by the Food and Drug Administration (FDA) as an orphan drug for the treatment of rare leukemias [15]. Our team revealed that Flavopiridol inhibits the activation of primary response genes, thus oppressing the down-stream inflammation of cells and tissues [9,10,11,12,13,14]. Therefore, Flavopiridol has been tested in the treatment of illnesses ranging from cancer [15,16,17] to viral [18,19,20] and post-traumatic-osteoarthritis (PTOA) [10,11,12]. Given its efficacy, Flavopiridol has been identified as a potentially promising candidate to treat illnesses such as lung cancer [21] and coronavirus disease 2019 (COVID-19) [22,23]. In most cases, anti-inflammation medicines were typically dosed systemically such as intravenous (i.v.) or oral delivery [24,25]. A high dosage and severe side effects often occurred due to systemic delivery [26]. Thus, local delivery would be more desirable such as pulmonary delivery by inhalation [27,28]. The local delivery of medicines to the lung is rapid in therapeutic efficacy and has been shown to improve the percentage of the bioavailable drug for treatment [29]. Additional advantages of inhalation delivery such as a simple dry powder inhaler (DPI) include non-invasiveness, high safety, and the rapid reaching of targets [30,31,32].

Inhalable particles are typically produced using a spray-drying technique, involving the rapid drying of liquid formulations into dry powders that are immediately ready for use [27,29,33]. While it produces high throughput, spray-drying uses a large quantity of materials and yields particles with wide size distribution. Thus, using spray-drying in research laboratories to carry out initial research and development (R&D) efforts, such as optimization of formulation and particle size, and understanding the underlying mechanism, is challenging due to the wide size distribution, time required to learn operation, and relatively high cost. This work introduces a simple home-constructed table-top device that enables the production of Flavopiridol-loaded inhalable ultra-small particles for pulmonary delivery. The particles produced meet the requirements needed for pulmonary delivery, including the physio-chemical property, inhalability, and release profiles. The anti-inflammation activity has also been demonstrated via in vitro testing. The device and conditions introduced in this work could be utilized by researchers in their R&D efforts in drug delivery. The particles produced will be tested in vivo for treatments of lung inflammation, and as a new therapeutic means to treat lung cancer, pulmonary fibrosis, and COVID-19.

## 2. Materials and Methods

### 2.1. Materials

1,2-dipalmitoyl-sn-glycero-3-phosphocholine (DPPC) was purchased from Avanti Polar Lipids (Birmingham, AL, USA). Flavopiridol was purchased from Cayman Chemical Company (Ann Arbor, MI, USA). L-leucine, L-isoleucine, D-(+)-Glucose, Calcium chloride (CaCl_2_), Tween-20, and Ethanol 100% were purchased from Sigma Aldrich (St. Louis, MO, USA). Phosphate-buffered saline (PBS) (1X) was purchased from Mediatech (Manassas, VA, USA). Gaseous nitrogen was purchased from Praxair (Danbury, CT, USA). Poly (tetrafluoroethylene) (PTFE) tubing was purchased from Cole-Parmer (Vernon Hills, IL, USA), and 96 well plates, 10- and 20-mL glass scintillation vials, and 1.00 mm ID glass capillary tubes were purchased from Fisher Scientific (Hampton, NH, USA). The variable area flowmeter was purchased from Dwyer Instruments Inc (Michigan City, IN, USA), and Dulbecco’s modified Eagle’s Medium (DMEM), Fetal Bovine Serum (FBS), and 2X Lysis reagent were purchased from Invitrogen (Waltham, MA, USA). Human embryonic kidney 293 cells (HEK-293) were purchased from ATCC (Manassas, VA, USA). Nuclear factor kappa B (NF-κB) reporter (cat # H-60650) and One-Step Luciferase Assay System were purchased from BPS Bioscience (San Diego, CA, USA). Tumor necrosis factor alpha (TNF-α) was purchased from Peprotech (Cranbury, NJ, USA). Ultrapure water with a resistivity of 18.2 MΩ·cm was generated using a Millipore Milli-Q system (EMD Millipore, Billerica, MA, USA). Gaseous nitrogen and gaseous CO_2_ were purchased from Praxair (Danbury, CT, USA).

### 2.2. Measurements of Loading Capacity and Efficiency of Flavopiridol-Encapsulated Inhalable Particles

Flavopiridol loading capacity and efficiency were determined using similar methods reported previously [14]. First, ~4.8 mg of particles ($m_{p}$) was dispersed in 1 mL of 70% (*v*/*v*) ethanol solution. The dispersion was sonicated for 15 min until complete dissolution of the particles was complete. The dissolved solution was loaded in a 1 mL syringe and filtered into a quartz cuvette using a 0.2 μm PTFE syringe filter (Sigma Aldrich, St. Louis, MO, USA). The filtered solution was subject to UV-Vis spectroscopy using a Denovix DS-11 spectrometer (Denovix Inc., Wilmington, DE, USA), and the absorbance (A) at 358 nm, characteristic of Flavopiridol, was measured. The molar extinction coefficient (ϵ) of Flavopiridol at 358 nm was determined to be 10,826 $M^{-1}{\mathrm{cm}}^{-1}$, using a standard series with concentration ranging from 3–100 μM in 70% (*v*/*v*) ethanol/water. Then, the concentration of Flavopiridol (c) was calculated using Beer’s law. The encapsulated mass of Flavopiridol ($m_{f})$ in the particles was determined by c × volume × the molar mass of Flavopiridol. The loading capacity (LC) of Flavopiridol was quantified using Equation (1):

(1) $$\mathrm{LC}=\left(\frac{m_{f}}{m_{p}}\right)\times 100\%\;$$

The encapsulation efficiency (EE) of Flavopiridol was quantified using Equation (2) by dividing the LC with the initial loading capacity in the organic phase, also known as the desired loading capacity of Flavopiridol, in this case, ${\mathrm{LC}}_{\mathrm{desired}}$ = 0.192% (*w*/*w*).

(2) $$\mathrm{EE}=\frac{\mathrm{LC}}{{\mathrm{LC}}_{\mathrm{desired}}}\times 100\%\;$$

### 2.3. Scanning Electron Microscopy Imaging of Produced Particles

The microparticle geometry and size were determined using a field emission scanning electron microscopy (SEM) (S-4100T, Hitachi High Technologies America, Inc., Pleasanton, CA, USA). Particles were immobilized to a double-sided carbon tape (Ted Pella Inc, Redding, CA, USA) supported by an Al stub (Ted Pella Inc. Redding, CA, USA). After coating the particles with 10 nm of gold using a sputter coater (208 HR High Resolution Sputter Coater, Ted Pella Inc., Redding, CA, USA), the samples were transported to the SEM vacuum chamber. The typical acceleration voltage and emission current used in this work were 2 kV and 10 μA, respectively. The SEM images were loaded into Image J (National Institutes of Health, Bethesda, MD, USA), where the size of the microparticles was determined using the geometrical Feret diameter ($d_{g}$).

The aerodynamic diameter ($d_{a}$) was calculated using Equation (3), following published method [32]. The tapped density (σ) was measured using established protocols [32], where the particle containers were tapped on a hard surface for 1000 taps. The reference density (ρ) is 1 g/mL, which is the standard reference for the diameter of a spherical particles that has the same vertical velocity as the particle of interest [34].

(3) $$d_{a}=d_{g}\sqrt{\frac{\sigma }{\rho }}$$

### 2.4. In Vitro Release Profiles of Flavopiridol from Inhalable Microparticles

In vitro release experiments were carried out to determine Flavopiridol release from the inhalable particles utilizing demonstrated release protocols [14,35]. Briefly, 5 mg of particles was placed in a 1.5 mL Eppendorf tube containing 0.5 mL of 1X PBS, and 0.2 (*v*/*v*)% Tween-20 was added to establish sink conditions. The particle dispersion was heated to 37 °C, to mimic physiological conditions. At each time point, the particles were centrifuged to a pellet at 12,000 rpm for 10 min, the supernatant was collected and filtered using a 0.2 μm PTFE syringe filter, and the tube was discarded. The supernatant was analyzed using UV-Vis spectroscopy at 358 nm absorption for the determination of Flavopiridol concentration. For all other time points, individual tubes containing particles were utilized. After all the tubes were analyzed for the release of Flavopiridol, the cumulative percentage of Flavopiridol was calculated using Equation (4), by taking Flavopiridol released up to a designated time, t, and then normalizing by the amount of Flavopiridol loaded ($m_{f}$).

(4) $$\mathrm{Cumulative}\;\mathrm{Release}\;\left(\%\right)=\frac{m_{t}}{m_{f}}\times 100\%$$

The in vitro release kinetics were quantified by fitting the measured profile using a 2-term model established previously [36,37], as illustrated in Equation (5):

(5) $$\frac{M_{t}}{M_{\infty }}=\theta_{b}\left(1-e^{-k_{b}t}\right)+\theta_{d}\left(1-\frac{6}{\pi^{2}}\overset{\infty }{\underset{n=1}{\sum }}\exp\left(-\frac{\pi^{2}n^{2}D_{e}t}{r_{g}^{2}}\right)\right)$$

The first term represented the process of “burst release”, with $k_{b}$ as the burst constant and $\theta_{b}$ as the contribution of burst release. This burst process was mostly described as interfacial diffusion, where a drug that was located near or at the surface of the particle underwent rapid dissolution [38]. The 2nd term describes the diffusion of drugs from spherical matrices under Fickian diffusion, where $D_{e}$ was the effective diffusion coefficient, $r_{g}$ was the geometrical mean particle radius, and $\theta_{d}$ was the contribution of diffusion release [39,40,41]. The constraint, $\theta_{b}+\theta_{d}=1,$ was used for mathematical completeness, representing individual contributions in the release mechanism. The kinetics parameters were attained by nonlinear least squares fitting (MATLAB, Math Works, Natick, MA, USA) of the measured release profile via Equation (5).

### 2.5. Stability Measurements of the Inhalable Particles

These particles were subject to exposure to three different environments. The first environment was set to 4 °C under ambient atmosphere, to mimic household refrigeration [42,43]. The second environment was to ambient atmosphere at 24 °C, representing household shelfs. The final environment was in an incubator set at 37 °C and 5% CO_2_ to represent a near physiological environment **[42,43]**. For each exposure condition, six 1.5 mL Eppendorf tubes were used, each filled with 1–2 mg of freshly prepared particles under ambient laboratory environment, 24 °C with relative humidity (RH) typically read at 40%. After exposure to the environment to a designated time, a vial was taken out, to which a 0.5 mL of 70% ethanol was added and then sonicated for 15 min until complete dissolution was achieved. The solution was filtered through via a 0.2 μm PTFE syringe filter and then collected into a quartz cuvette for the UV-Vis measurements. The measured spectrums were compared against that of freshly produced particles to determine aging and stability.

### 2.6. Attenuated Total Reflectance—Fourier Transform Infrared Spectroscopy

Attenuated Total Reflectance—Fourier Transform Infrared Spectroscopy (ATR-FTIR) [44] was used to characterize the particle formulation for the identification of components, including excipients and drugs. Briefly, <1 mg of freshly produced formulation particles was placed and compressed on the ATR diamond crystal. The FTIR spectrum was acquired using a Bruker Tensor 27 FTIR (Bruker Corporation, Billerica, MA, USA). The spectrum was obtained over wavenumber range, 4000–400 ${\mathrm{cm}}^{-1}$. The spectrum was averaged over 30 scans with a wavenumber resolution of 4 ${\mathrm{cm}}^{-1}$. The individual components of the formulation particles such as Flavopiridol, DPPC, and L-isoleucine were acquired for comparison as a control.

### 2.7. In Vitro Bioactivity Assay

The anti-inflammation activity of Flavopiridol released from inhalable particles was determined by a luciferase reporter assay as described previously [14,45]. Briefly here, sufficient microparticles were dissolved in DMEM with 10% FBS to a stock solution of 8.9 × $10^{4}$ nM Flavopiridol. This concentration was further diluted 297X to the final therapeutic concentration of 300 nM Flavopiridol [9]. The bioactivity of Flavopiridol was measured by its ability to suppress TNF-stimulated luciferase reporter expression, driven by a NF-kB responsive promoter. The HEK293 cells harboring a NF-kB-driven luciferase reporter were seeded in 96-well plates (10,000 cells/well in triplicates) 24 h before the experiment. Cells were then treated with 100 μL of media containing 0.6 nM recombinant human TNF-α, in the presence or absence of dissolved Flavopiridol formulation or blank formulation equivalent. After 24 h, 100 μL of 2X lysis buffer containing luciferase substrate was added directly to each well. Luminescence was measured in a plate reader (SpectraMax iD3, Molecular Devices, San Jose, CA, USA).

### 2.8. Aerodynamic Performance Testing

The aerodynamic performance studies followed the pharmaceutical industry’s good practice and were carried out by iPharma Labs, Inc (Union City, CA, USA). The emitted powder mass (EPM) was measured from the emitted aerosol dose from a RS01 dry powder inhaler (DPI). Briefly, 5 mg of inhalable particles was filled into size 3 clear capsules and loaded ($m_{\mathrm{loaded}}$) into the DPI. The aerosol mass dose emitted ($m_{\mathrm{emitted}})$ from the DPI was collected on a custom-delivered dose apparatus (iPharma Labs Inc, Union City, CA, USA) with a 81 mm diameter glass fiber filter (Pall Corp, Port Washington, NY, USA). The testing air-flow was generated using a timer-controlled solenoid valve connected to a vacuum source. The flow rate was adjusted using a flow control valve. The volume of sampled air was maintained at 2 L for each dose actuation for EPM. When actuated with flow, the aerosolized dose leaving the inhaler mouthpiece was deposited onto an 81 mm filter (Type A/E, Pall Corp, Port Washington, NY, USA) and housed within custom filter holders, designed for engineered particles. The larger 81 mm diameter filter was used to minimize filter pressure drop for the RS01 device, which has a medium flow resistance, corresponding to 4 kPa pressure drop for RS01 having a flow resistance of (cm H_2_O)^1/2^ min/L. The EPM is calculated below, as seen in Equation (6):

(6) $$\mathrm{EPM}=\frac{m_{\mathrm{emitted}}}{m_{\mathrm{loaded}}}\times 100\%$$

The inhalable particles were delivered from an RS01 DPI, and the aerodynamic performance was tested using a next-generation impactor (NGI) (USP,601. Apparatus 5) using a modified NGI gravimetric cups (P/N 5241 and 5244, MSP Corp, Shoreview, MN, USA), fitted with 55 mm glass fiber filters (P/N GB-100R, Advantec MFS Inc, Dublin, CA, USA) as impaction surfaces. The NGI set up consisted of seven stages and a micro-orifice collector (MOC). An internal filter for the MOC stage (P/N 5206, MSP Corp, Shoreview, MN, USA)) with a 76 mm glass fiber filter (P/N 61663, Pall Corp, Port Washington, NY, USA) was used in all test runs. For each test run, two capsules (Size 3, clear) were filled with 5 mg of inhalable particles. Each capsule was loaded into the RS01 device, and an aerosolized mass was collected by the NGI at 60 L/min, which corresponds to 4 kPa drop for RS01 having a flow resistance of 0.10 (cm H_2_O)^1/2^ min/L.

The powder mass collected on filters for both EPM and NGI tests was measured by gravimetric analysis due to their homogeneous compositions and reported in terms of % of the nominal fill mass [46]. The NGI recovered mass ($m_{\mathrm{recovered}}$) was determined by the gravimetric analysis of the stages 1–7 and MOC with respect to the actuated inhalable particles from DPI. The fine particle fraction (FPF) was quantified by the % fraction of the emitted dose deposited on stage 2—MOC with an aerodynamic diameter ($d_{a},NGI)$ ≤ 5 μm. The mass median aerodynamic diameter (MMAD) was the median $d_{a,\;NGI}$ of the emitted dose at the 50% mark of the cumulative fraction with respect to the cut-off diameter of the NGI stage. The geometric standard deviation (GSD) measured the ratio of the particle diameter at 84% and 16% of the cumulative distribution curve, which represents the variability or breadth of the particle size distribution [44,47].

### 2.9. Moisture Content

The moisture content of the produced inhalable particles was quantified by iPharma Inc. (Union City, CA, USA) using Karl Fischer titration on a coulometric Nittoseiko Analytech Moisture Meter CA-310 with a frit-less cathode and Vaporizer VA-300 (Nittoseiko Analytech Co., Ltd., Yamoto, Japan). In a glove box, 60 mg of inhalable particles was loaded into a capped 4 mL glass vial (VWR International, Radnor, PA, USA) and then analyzed at 100 °C in an oven. Measurements of the inhalable particle moisture content were performed in triplicate.

## 3. Results and Discussions

### 3.1. A Home-Constructed Table-Top Microdevice for Synthesis of Inhalable Microparticles

**Figure 1:**
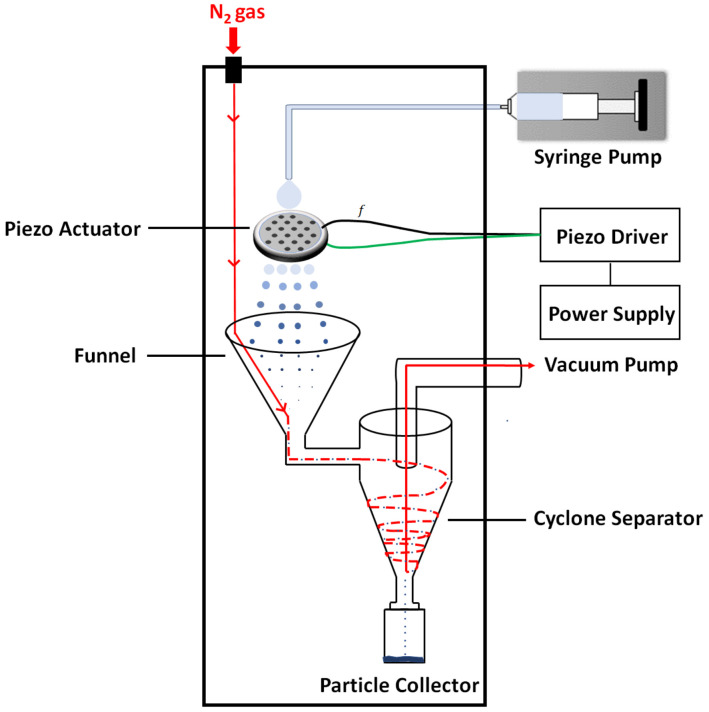
Schematic diagram of our table-top microdevice for the synthesis of inhalable particles.

The home constructed table-top microdevice (TTMD) was illustrated schematically in Figure 1. The entire device was enclosed inside a custom acrylic box, 75 cm × 75 cm at the base and 100 cm tall. The TTMD, including the enclosure, weighs less than 50 lbs, which is very portable and easily fitted into a research laboratory environment. The mechanical parts were designed using Solidworks^®^ CAD software (Dassault Systèmes SE, Vélizy-Villacoublay, France). A 50 mL plastic BD syringe (Becton, Dickinson and Company, Franklin Lakes, NJ, USA) served as a holder for the feed solution, whose back was pushed by a syringe pump (NE-1000, New Era Pump Systems, Inc, Farmington, NY, USA). The solution exited to a 23 AWG PTFE tubing tightly connected to a 22 AWG blunt needle (Component Supply Co, Fort Meade, FL, USA). 2 cm below the needle was a stainless steel (SS) plate with an array of orifices (SMMOD20F113H18, STEINER and MARTINS, INC., Davenport, FL, USA). The two piezo rings were fixed atop and at the bottom of the SS plate, respectively, serving as the actuator. The piezo actuator was controlled by our home-constructed piezo driver at 10–150 kHz and 0–30 V amplitude, and 50 cm below the plate, a custom PTFE funnel (D = 21 cm opening) served as the initial liquid droplet collector, with the narrow exit connect to our custom cyclone chamber (10 cm top diameter, 20 cm in length). The cyclone separator adopted a cylinder-on-cone design with a tangential inlet and was manufactured in PTFE. The exit was threaded to a glass container to collect solid particles. The vacuum suction was from the top center of the chamber via a silicone tubing connecter to a mechanical pump (RV5, Edwards Vacuum LLC, San Jose, CA, USA).

During operation, the feed solution was pushed into the PTFE tubing through the syringe pump at a designated flow rate. The solution exited through the blunt needle showering ~1 cm^2^ area of the stainless steel (SS) plate with orifice array. The vibration of the plate interrupted the liquid flow into a spray of small droplets. By varying the aperture size and/or vibrational frequency, microfluidic devices enable the tuning of droplet size [48]. Using our set up, a droplet diameter ranging from several to tens of mm can be attained by tuning our experimental conditions. High-purity (99.99%) nitrogen gas was introduced through an inlet atop of the enclosure. The nitrogen flew through the funnel, into the chamber, forming a cyclone, and got pumped away, as indicated by the trajectory in Figure 1 (red line). The gas carried and dried the micronized liquid droplets along the pathway, leading to the precipitation of dry solid particles onto the walls of the chamber and at the bottom of the glass container. The formation of cyclone was essential to assure the sufficient travel path length of the liquid droplets to allow for the drying and formation of solid particles.

In this TTMD, the initial volume of liquid is up to 50 mL per batch, sufficient to produce up to 250 mg of particles per hour. A typical dosage of inhalable particles is around 5 mg for humans [49]; thus, each batch produces 50 doses for in vivo studies. The liquid flow rate is tunable from 5 to 100 mL/hr. Several SS plates were used for this work, with the designed diameters of the micro holes ranging from 4 to 11 μm, which manifested in liquid particle sizes ranging from 5 to 100 μm. The nitrogen gas flow rate could be varied from 5 to 100 mL/hr and the aperture-collector distance from 20 to 80 cm. Adding to the device shown in Figure 1, a high-speed camera (AX100, Photron Inc., San Diego, CA, USA) with a long working distance lens (96 mm) and magnification (2X) was mounted to visualize the droplets exiting the array. Videos were taken at 20,000 frames/sec during synthesis to allow us to change parameters to attain the designed droplet size. The liquid formulation and these tunable parameters have been optimized (see next section) to produce particles with a desirable size, morphology, and physio-chemical properties for the specific applications.

### 3.2. Determination of the Solution Formulation and Optimal Synthesis Conditions

As the initial estimation of Flavopiridol concentration in the solution before activating microfluidic synthesis, we examined the practical factors pertaining to human inhalation. Enroute to the lung for being uptaken, a portion of inhalable particles would reach the epithelial lung lining fluid (LLF), where gradually, Flavopiridol would get released and become soluble and thus bioavailable in LLF for human uptake [28]. A typical human LLF has a volume of ~20 mL [50], and a typical single puff from a DPI contains 5 mg of total particles [49]. To reach our targeted therapeutic Flavopiridol concentration of 300 nM in LLF, the loading capacity (LC) of our particles should at least be 0.048% (*w*/*w*) based on the definition of LC shown in Equation (7).

(7) $${\mathrm{LC}}_{\mathrm{desired}}=\frac{V_{\mathrm{LLF}}\times \left(C_{\mathrm{Therapeutic}}\;\right)\times \left({\mathrm{MW}}_{\mathrm{Flavo}}\right)}{m_{\mathrm{dose}}}\times 100\%$$

The additional consideration of LC includes the loss of the drug before reaching LLF. It was reported that nearly 25% of the encapsulated drug could reach the intended destiny in cases of pulmonary delivery using a DPI [51]; therefore, the targeted amount of Flavopiridol was increased to 4X, i.e., LC = 0.19%.

Besides drug dosimetry, further considerations include inhalability and non-aggregation for the resulting solid microparticles [28,52]. The physiochemical properties pertaining to these considerations include aerodynamic diameters 1–5 μm [27,53], fully dried (moisture content below 0.6%) [32], and highly corrugated surfaces with folded geometry to prevent clustering [33,54].

The next consideration is selecting solvents suitable for our TTMD; followed by selecting and varying the excipients and concentration of Flavopiridol to reach the desired particle morphology, and dispersion; and, finally, varying experimental parameters (see above) to reach the designed size for inhalation.

**Figure 2:**
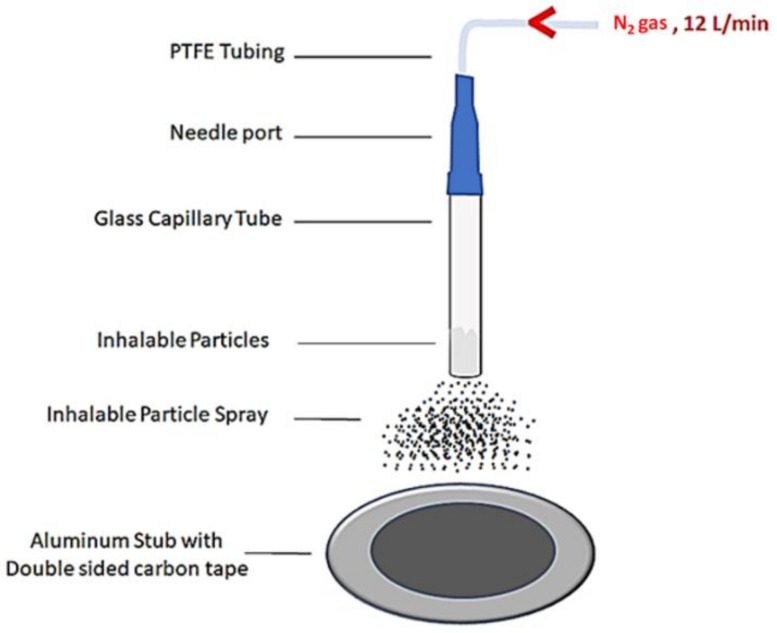
A simple air-flow particle disperser for depositing microparticles onto SEM sample holder for imaging and determining dispersibility.

For measurements of particle size and for visualizing particle morphology, our field-emission SEM was utilized. Unlike standard and conventional SEM imaging of drug delivery particles [14,44], our sample preparation attempted to mimic the situation of particles in airways during inhalation. A simple air-flow particle disperser (AFPD) was assembled, as illustrated in Figure 2.

Briefly, <1 mg of powder formulations was inserted into a 1.00 mm ID glass capillary tube and mounted vertically via three-fingered clamps. The end of the capillary tube was custom-fitted with a syringe-needle port (Blunt 20 AWG, Component Supply Co., Fort Meade, FL, USA), which was inserted into a 21 AWG PTFE tubing. The end of the PTFE tubing was directly fitted to a variable area flowmeter (Dwyer Instruments Inc, Michigan City, IN, USA) attached to nitrogen gas tank. The flow rate of nitrogen was set at rates comparable to the nominal human breathing at sea level, 6–16 L/min [55]. The flow of nitrogen gas through the glass capillary tube reached the particles and guided them out of the bottom of the tube for 8 cm until striking a double-sided carbon tape (Ted Pella Inc, Redding, CA, USA) fixed onto an Al stub (Ted Pella Inc., Redding, CA, USA). Upon completion of sample preparation, the samples were coated with 10 nm of Au and then transferred to the vacuum chamber for SEM imaging, as described in the experimental section.

To assure complete solvent evaporation using our TPMD, a mixed solvent of ethanol:water with volume ratio ranging from 50:50 to 90:10 was tested. The purpose was to assure the solubility of Flavopiridol and of all excipients in a single phase in the beginning to attain dry particles at the collector upon completion of synthesis. The optimized ratio for this work was determined to be ethanol:water = 70:30.

**Figure 3:**
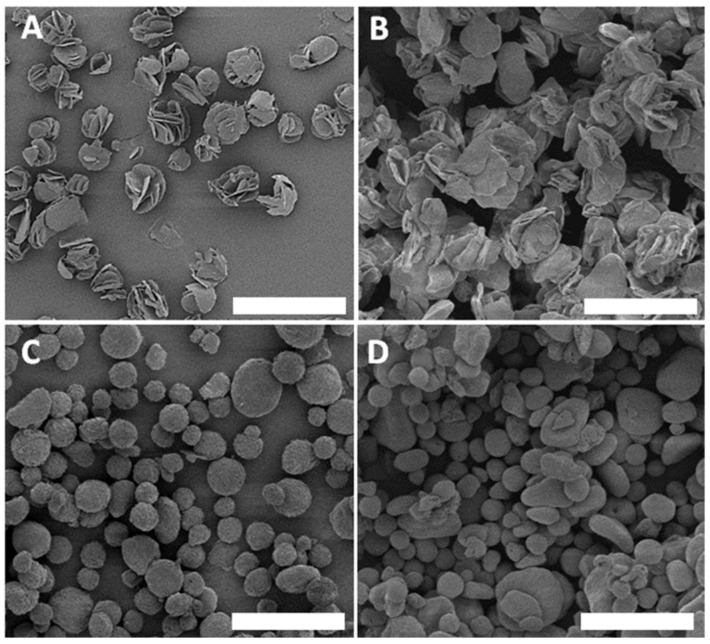
SEM imaging from four experiments during our investigations to determine our formulation. (**A**) Excipients include L-isoleucine:DPPC = 90:10 (*w*:*w*) in a mixed solvent of 70% (*v*/*v*) ethanol/water. (**B**) L-isoleucine:DPPC:CaCl_2_ = 89:10:0.75 in a mixed solvent with the same mixing as that in (**A**). (**C**) L-isoleucine:DPPC:Glucose = 10:10:80 in a mixed solvent with the same mixing as that in (**A**). (**D**) L-isoleucine:DPPC:Glucose = 10:10:80 in a mixed solvent of 80% (*v*/*v*) ethanol:water. Scale bars = 20 μm.

In the design of inhalable particles, excipients or non-active components represent major components of the particle, thus dictating the physio-chemical properties and inhalability of the particles [31,32]. Many excipients have been used in inhalable particle formulations, such as amino acids, sugars, and lipids [29,44,56,57,58]. Amino acids such as L-leucine and L-isoleucine are frequently used due to their high biocompatibility and unique property as dispersibility enhancers [32,57]. During initial investigations, both L-Leucine and L-isoleucine were tested as the primary excipients in the formulation. L-isoleucine was found to be preferable due to its higher solubility in our chosen solvent. The addition of a minute number of phospholipids to the formulation has been known to facilitate particle entry into cells [59,60]. In this investigation, 1,2-dipalmitoyl-sn-glycero-3-phosphocholine (DPPC) was the chosen lipid, following a recommendation from prior excipient formulations used in pulmonary delivery of other medicine [35,44]. In addition to the known benefits, we found that the addition of a phospholipid to our fed solution led to a rough particle morphology, as shown in Figure 3A, where outward folded sheets analogous to a “rose-bud”, which is a preferred morphology for pulmonary delivery due to high surface area, thus enhanced particle–lung-fluid interactions and reduced agglomeration [61,62]. Various other excipients were also tested. It has been shown with the commercial product, Pulmosphere, that the addition of (2:1) CaCl_2_ to phospholipid formulations resulted in improved environmental robustness [63]. As seen in Figure 3B, the addition of CaCl_2_ resulted in heterogeneity in shape with a flattened heterogenous morphology; therefore, it was not beneficial for this synthesis. The addition of sugars, such as glucose, has been utilized in prior synthesis as an enhancer for bioavailability during pulmonary delivery [56,64]. In our case, the addition of glucose led to the particles shown in Figure 3C,D, which exhibited a lower surface-to-volume ratio than that in the L-isoleucine containing particles. Taken collectively, the excipient components for our particles are L-isoleucine:DPPC = 90:10 in *w*:*w*.

Assuming all components in the solution are equal to the final composition of the particles, the final formulation would be L-isoleucine:DPPC:Flavopiridol = 89.8:10.0:0.19. The typical total concentration used in spray drying was in the range of 0.1–0.5% (*w*/*v*) [31]. In this work, we tested the full range 0.1–0.5% and determined the optimal concentration to be 0.3%. Based on our chosen formulation, we refer to these L-isoleucine:DPPC:Flavopiridol microparticles as ILDF.

Finally, with the formulations at hand, we varied the TTMD operation parameters systematically until the geometrical diameter of the particles was around 5–6 μm and the designed morphology (Figure 3A) and high dispersity were achieved. The testing range and final parameters were summarized in Table 1.

Under the specified conditions (Table 1) to produce ILDF, the humidity inside the TTMD enclosure measured 25%, while the exit droplet measured 12 ± 3 mm via our highspeed-camera through a long working-distance lens. These measurements are consistent with the anticipated liquid droplet size estimated by manufacturer (18 μm). The moisture content of the ILDF particles was measured and summarized in Table 2, column 5. The moisture levels for all three batches of our samples were 0.52 ± 0.01% (*w*/*w*), which is lower than other reported inhalable particles [65] and comparable with similar excipients like DPPC and L-leucine [44].

**Figure 4:**
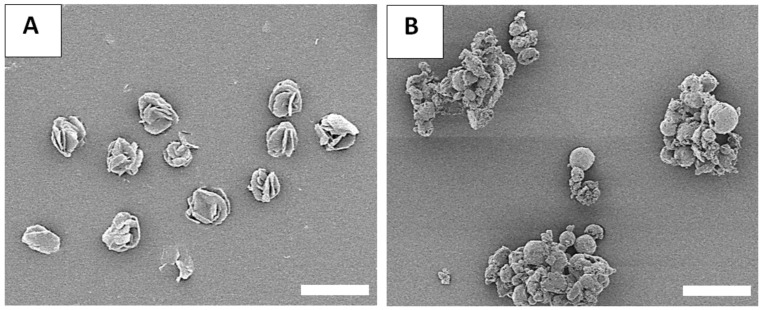
High-resolution SEM imaging of (**A**) ILDF particles used for our inhalation delivery; and (**B**) microparticles formed from 0.3% solution of L-isoleucine:DPPC:Glucose = 10:10:80 using 70% (*v*/*v*) ethanol:water. All scale bars = 20 μm.

The size, morphology, and dispersity of the optimized particles were determined via SEM and are shown in Figure 4A. The ILDF particles exhibit high dispersity, with a mean distance between particles ($\bar{x}$) = 7.3 ± 5.5 μm. In comparison, microparticles with 80% D-α-glucose were imaged and shown in Figure 4B. In Figure 4B, ILDG particles are clumped together into aggregates of at least 20 particles, indicating low dispersibility. Our SEM studies support prior knowledge that sugars at high compositions are sticky and adhesive and thus tend to become clustered [56]. The diameter of ILDF particles measured from SEM images is 5.5 ± 1.3 μm. This diameter is known as the geometrical diameter (summarized Table 2, row 2). The bulk/tapped density of the produced ILDF particles was measured to be 0.2 ± 0.01 mg/mL. The aerodynamic diameter was calculated using Equation (3) to be 2.5 ± 0.6 μm, meeting the requirements of $d_{g}$ = 1–5 μm for pulmonary delivery [27]. These ILDF particles exhibited high dispersity, with a mean distance between particles ($\bar{x}$) = 7.3 ± 5.5 μm. As a comparison, microparticles with 80% D-α-glucose were imaged and shown in Figure 4B. In the formulation used in Figure 4B, the microparticles were clustered into aggregates of at least 20 particles per cluster, indicating low dispersibility. Our observations are consistent with prior knowledge that sugars at high compositions were sticky and adhesive, thus easily becoming clustered [56].

**Figure 5:**
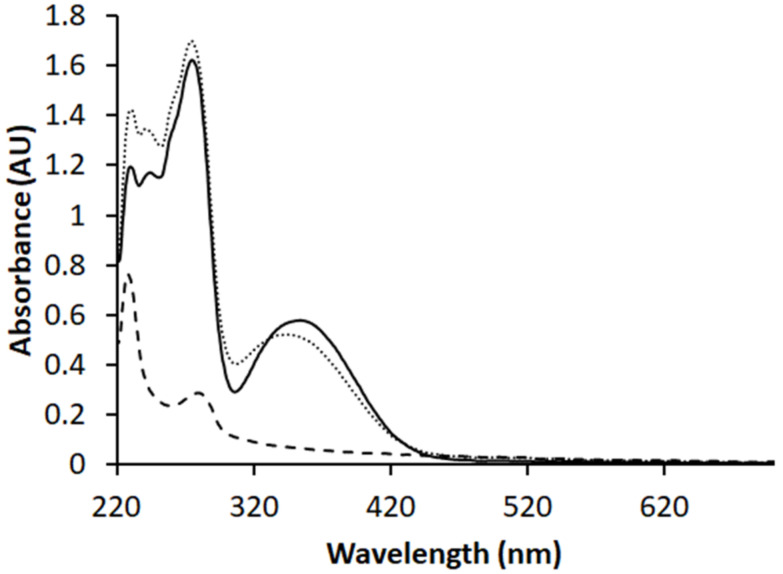
Absorption spectra of ILDF microparticles after dissolving in 70% ethanol (short dash) solution, displayed with that of 50 μM Flavopiridol in 70% ethanol (solid black line). The spectrum of the same microparticles without Flavopiridol, i.e., ILD microparticles, serves as blank or negative control and is plotted in the same coordinate (long dash) as a comparison.

To verify that the active component, Flavopiridol, retained its structural integrity and chemical functionality in our TTMD synthesis, we measured the UV-Vis spectra for Flavopiridol as received, and that from fully dissolved ILDF particles. The similarity of the two spectra, shown in Figure 5, demonstrates that the chemical integrity or functionality of Flavopiridol were clearly preserved.

The Flavopiridol concentration in ILDF can be quantified from the spectra at $\lambda_{max}$ = 358 nm. This peak was selected because there is no overlap with any other components such as in the spectrum of ILD microparticles (see long dash in Figure 5). Following the protocol in the experimental section, the molar extinction coefficient for Flavopiridol is calibrated to be 10,826 $M^{-1}{\mathrm{cm}}^{-1}$, with an absorbance of ILDF solutions measured A = 0.5003. Thus, the resulting Flavopiridol concentration is 4.621 × $10^{-5}$ M, and therefore the loading capacity of Flavopiridol in the ILDF particles was quantified using Equation (1) to be LC = 0.19%. The encapsulation efficiency (EE) in this synthesis was quantified using Equation (2) reached 99 ± 2%. The overall yields, i.e., products/starting materials, consistently reached over 45%. The synthesis process was overall reproducible between batches as the LC and EE were calculated in triplicate from individual syntheses. The robustness was demonstrated by increasing the total feed solution concentration to produce larger particles. Therefore, the synthesis efficiency of our TPMD is very consistent and high.

**Figure 6:**
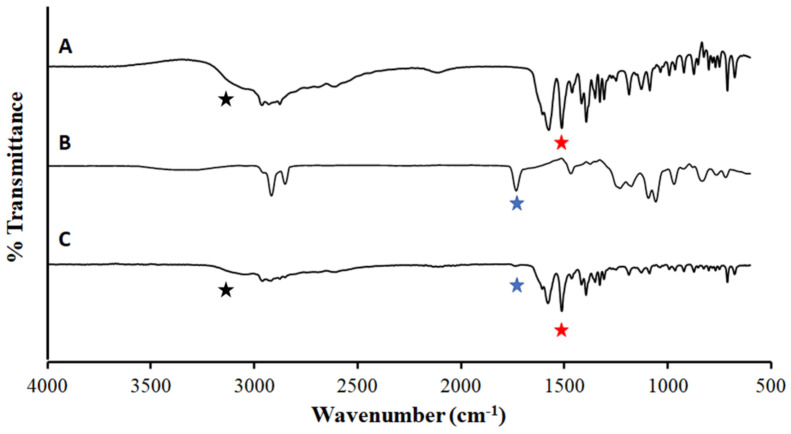
ATR-FTIR spectra of excipient materials of (**A**) L-isoleucine, (**B**) DPPC, and displayed in conjunction with the ILDF microparticles (**C**).

To check and verify the chemical integrity of other components in our ILDF microparticles, ATR-FTIR spectroscopy was acquired. As shown in Figure 6, the spectrum of ILDF is compared with that of individual components of L-isoleucine and with DPPC. The spectrum of the ILDF primarily resembles that of L-isoleucine (A), as evidenced by the characteristic N-H bending, 1514 ${\mathrm{cm}}^{-1}$ (red star) [66], and the N-H stretching 3200 ${\mathrm{cm}}^{-1}$ (black star) [67]. The presence of DPPC is also clearly proven by the well-resolved C=O stretching mode, 1700 cm^−^^1^ [68] (blue star). The intensity is consistent with the fact that L-isoleucine was the major component of our formulation (89.9%), while DPPC (10%) is a minor component.

### 3.3. Aerodynamic Performance of the ILDF Particles

**Figure 7:**
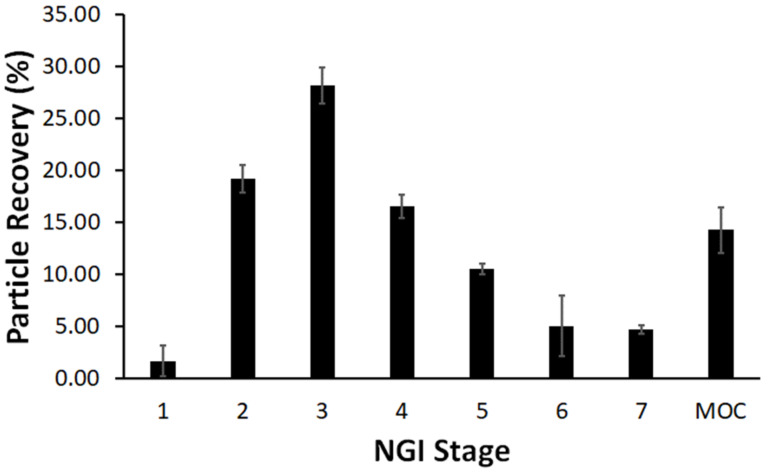
NGI stage fractioning of ILDF particles from a RS01 DPI at 60 LPM (*n* = 3).

The testing of the aerodynamic performance of our ILDF particles followed the industry’s standard of good practice and was carried out through measurements of EPM, MMAD, FPF, and GSD using an NGI from the emitted aerosol dose from a RS01 DPI. The EPM was measured from the emitted aerosol dose as the average of five capsules with nominal filled mass of 5 mg. The EPM was determined to be 96.5 ± 3.8%, which rivals (and exceeds) commercial products [69]. The measurement of MMAD, FPF (<3.3 μm), and GSD were determined from the distribution of fractioned particles in the NGI. The resulting graph of the particle % recovery with respect to NGI stage (*n* = 3) is shown in Figure 7.

The particle size fractionation distribution is observed in Figure 7, from stages 1-MOC. In accordance with USP <601>, the MMAD was determined from the intercept of the fitting of the lognormal distribution plot and was determined to be 2.7 ± 0.2 μm. This experimentally determined aerodynamic diameter is quite similar to the $d_{a}$ = 2.5 ± 0.6 μm (Table 2) calculated from the SEM imaging of our ILDF particles. The aerodynamic diameters of these ILDF particles are well within the optimal size range (1–5 μm) for pulmonary delivery [27,29,33]. The GSD was determined to be small with 2.3 ± 0.8, which indicates a narrow particle size distribution [44]. The FPF, d < 3.3 μm, was determined to be 61.6 ± 2.5%, which means the majority of our particles would reach the lung (d~1–5 μm). Overall, the total recovery of the emitted dose in the NGI was determined to be 58.9 ± 4.0%. These aerodynamic results are summarized below in Table 3. This outcome is very comparable to peer excipients such as L-leucine-based inhalable particles [70].

### 3.4. The Release Profile of Flavopiridol from Microparticles

**Figure 8:**
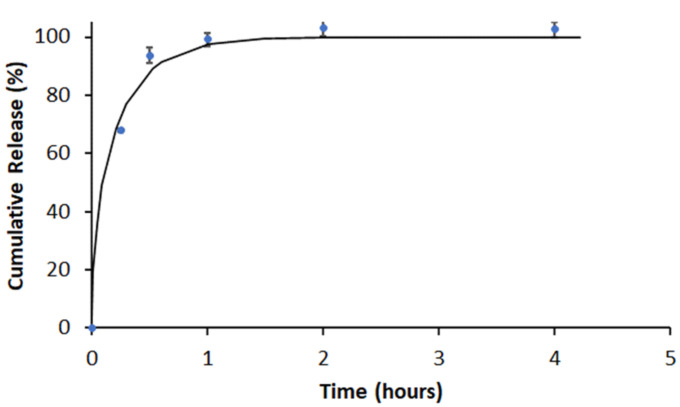
Flavopiridol released at designated times after soaking particles in 1X PBS with 0.2% (*v*/*v*) Tween-20 at 37 °C (blue dots). In each time point of the measurement, the result is expressed as mean ± SD from *n* = 3 independent batches of ILDF microparticles. Nonlinear least-square fitting of the release profile using Equation (5) is shown as the solid black line.

In vitro release experiments were carried out for the Flavopiridol loaded ILDF particles, utilizing the protocols described in Section 2.4 [14,35]. As shown in Figure 8 (blue dots), Flavopiridol was rapidly released from the 5.5 ± 1.3 μm ILDF particles, reaching ~70% at 0.25 hrs and 99.4% at 1.3 hrs.

The quantification of release kinetics follows the known kinetics model, described in Section 2.4. The non-linear least square fitting of the measured profiles using Equation (5) enabled the extraction of kinetics parameters as summarized in Table 4. The burst constant, $k_{b}$ = 1.599 ${\mathrm{hr}}^{-1}$, demonstrates how fast the Flavopiridol was released upon soaking. The diffusion coefficient was $D_{e}$ = 2.3 × $10^{-11}$ ${\mathrm{cm}}^{2}$/s, which was similar to those reported for other drug release studies [39]. This rapid release of Flavopiridol from our ILDF microparticles is desirable for inhalable drugs, analogous to the prior successful examples of inhalable insulin and budesonide [52,71].

### 3.5. The Inhalable Microparticles Are Sufficiently Stable

The ILDF particles (Table 2, row 2) were subject to stability studies [42,43] before our future work of animal tests and preclinical trials. First, the absorption of water was tested by weighting the ILDF particles for the durations of one week. The total mass remained the same as the freshly prepared particles within ±0.03 mg. Then, we exposed the particles to three designated environments. The first environment was set to 4 °C in an ambient atmosphere, to mimic household refrigeration [42,43]. The second environment was in an ambient atmosphere at 24 °C, 40% RH, representing household shelf conditions. The final environment was in an incubator, 37 °C and 5% CO_2,_ to represent a near physiological environment [42,43].

**Figure 9:**
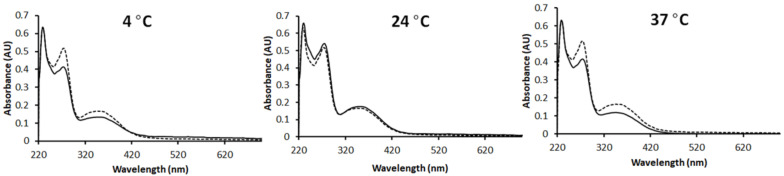
Left, Middle, and Right are the results of the stability tests of Flavopiridol in ILDF inhalable particles stored for up to 4 weeks at 4, 24, and 37 °C, respectively. The UV-Vis spectra of the ILDF microparticles in 70% ethanol (solid black line) after 4 weeks of storage is displayed with that of the freshly produced ILDF microparticles (dashed black line). The absorption intensity was normalized using λ = 230 nm to allow for easy display and comparison.

At 4 °C and after 4 weeks, as shown in Figure 9 (left), the UV-Vis spectrum of Flavopiridol from the ILDF particles is almost identical to that of freshly produced ILDF particles. Therefore, under refrigerated conditions the ILDF microparticles can be kept for at least a month. At room temperature 24 °C (Figure 9, middle), similar stability was observed. Even at an elevated temperature, these ILDF microparticles remained unchanged within our test period of one month.

**Figure 10:**
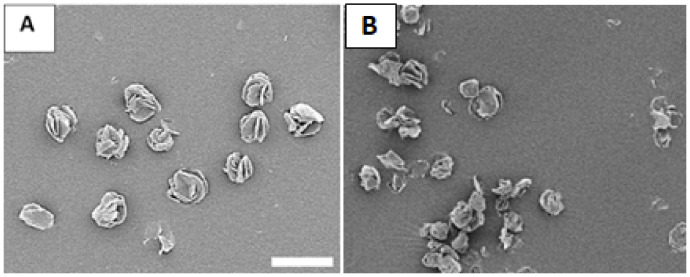
SEM images of the ILDF particles (**A**) freshly prepared; and (**B**) after 24 weeks of exposure in ambient conditions. Scale bar is 20 μm.

To test the physical stability and structural integrity of the produced ILDF particles, the particles subject to the harshest exposure conditions among the three, i.e., 40% RH, 24 °C, and ambient laboratory conditions, were imaged using SEM. Figure 10 shows the results from freshly prepared microparticles in comparison to that after 6 months of exposure. The freshly produced ILDF particles seen in Figure 10A present a characteristic “Rose-bud” morphology with 4–5 outward folds per particle. These particles are well dispersed. After 24-weeks stored under ambient conditions, the ILDF particles still exhibited the characteristic “Rose-bud” morphology, as seen in Figure 10B. About 50% of particles became fractured, forming clumping or smaller particles.

These results are very encouraging, well positioning our products as candidates for further investigation, including in vivo and preclinical testing.

### 3.6. Flavopiridol Encapsulated Microparticles Exhibit High Anti-Inflammation Activity

**Figure 11:**
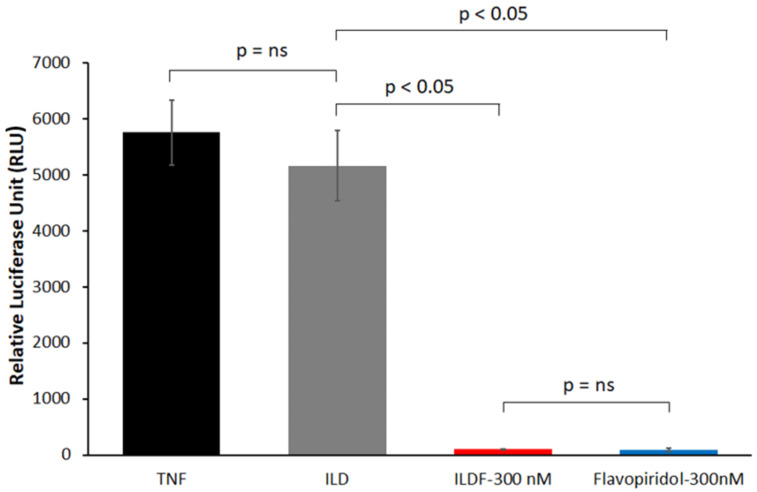
Bioactivity of Flavopiridol in ILDF particles measured by luciferase assay. Released Flavopiridol from ILDF particles and blank ILD particles were assessed by measuring the suppression of luciferase activity in HEK-Luc reporter cells stimulated with TNF-α (0.6 nM). Experiments were performed in triplicate (N = 3), and results were reported as mean ± SD.

The biological activity of Flavopiridol in the ILDF particles was assessed using a luciferase assay [14,45]. The presence of TNF-α in the luciferase assay causes the activation of luciferase expression, which is driven by the NF-κB promoter. In the presence of Flavopiridol, this activation is suppressed. As seen in Figure 11, The maximum luciferase activity was detected in cells treated with TNF only (black bar), as expected, because there was no inhibitor present. The blank ILD particles (Grey bar) showed similar behavior with no statistical difference because the luciferase expression continued uninterrupted without Flavopiridol.

In comparison, as a positive control, cells were treated with 300 nM of soluble Flavopiridol (blue bar). It is clear that the luciferase activity became oppressed dramatically, due to the presence of Flavopiridol, limiting the luciferase expression. The performance of the Flavopiridol in the ILDF particles (red bar) matched the positive control with little statistical difference. These measurements suggest that the biological activity of Flavopiridol released from the ILDF particles was retained. These tests paved the way for further investigations of pulmonary delivery in vivo in the near future.

## 4. Conclusions

A table-top microdevice was designed and constructed to produce ultrasmall particles for drug delivery via inhalation. The design and operation were very similar to that of a spray-drying equipment used in industry, but the device itself is much smaller and portable in size, more accurate in particle size control, and simpler to operate and economical. Using Flavopiridol as an anti-inflammation medication, formulations of the initial liquid were developed. The liquid formulation droplets passed through the micro-aperture arrays of the device that was shaken via a piezo-electrical driver. High-purity (99.99%) nitrogen gas was introduced and flew through the designed path, including the funnel collection and the cyclone chamber, and finally was pumped away. The gas carried and dried the micronized liquid droplets along the pathway, leading to the precipitation of dry solid microparticles. The formation of cyclone was essential to assure the sufficient travel path length of the liquid droplets to allow for the drying and formation of solid particles. Synthesis parameters have been optimized to produce microparticles, whose morphology, size, physio-chemical properties, and release profile met the criteria for inhalation. The aerodynamic testing, including MMAD, FPF, and GSD determination, concluded that the ILDF particles revealed excellent aerodynamic performance. Bioactivity assays have revealed a high degree of anti-inflammation. The advantages are clearly demonstrated, including a high degree of consistency in particle size and composition and simplicity in device fabrication and usage. Thus, this approach enables the production of inhalable particles in research laboratories in general. The generality of the microdevice also allows for the production of a wide range of drugs beyond Flavopiridol, including Atuveciclib [72], LDC000067 [73], and CAN508 [74]. Other classes of molecules could be formulated into inhalable particles to meet application needs, such as carbohydrates, proteins, peptides, and oligonucleotides. For industrial and high throughput productions, a larger scale device would be necessary, for example, modified spray drying equipment. Work is currently in progress to test the anti-inflammation efficacy of these inhalable particles using animal models (rodents). Built upon animal models, future clinical applications are envisioned, including rapid treatments of lung inflammation and pertaining diseases, such as pulmonary fibrosis and COVID-19.

## Figures and Tables

**Table 1:** The range and optimized TTMD operation parameters for particle synthesis.

Parameter	Range	Value
Micro-piezo orifice diameter	4–11 μm	11 μm
Feed solution temperature	23 ± 1 °C	23 ± 1 °C
Feed solution flow rate	5–30 mL/hr	22.0 mL/hr
$N_{2}$ gas flow rate	5–30 L/min	15 L/min
$N_{2}$ gas temperature	23 ± 1 °C	23 ± 1 °C
Piezoelectric driving frequency	10–150 kHz	113 kHz

**Table 2:** Summary of the ILDF microparticles in comparison to the control (ILD) produced using our TTMD. Basic properties of these particles, including moisture content, $d_{g}$, $d_{a}$, and dispersion, are included.

Formulation	Process Yield (%)	LC (%)	EE (%)	Moisture Content (%(*w*/*w*))	$\rho_{\mathrm{tapped}}\;(\mathrm{mg}/\mathrm{mL})$	$d_{g}$ (μm)	$d_{a}$ (μm)	Dispersion
ILD	43	0	0	-	0.19 ± 0.01	5.4 ± 1.2	2.4 ± 0.5	High
ILDF	45	0.19 ± 0.01	99 ± 2	0.521 ± 0.013	0.20 ± 0.01	5.5 ± 1.3	2.5 ± 0.6	High

**Table 3:** Summary of aerodynamic performance testing of the ILDF particles.

Sample	EPM (%)	FPF, <3.3 μm(%)	MMAD (μm)	GSD	NGI Recovery (%)
ILDF	96.5 ± 3.8	61.6 ± 2.5	2.7 ± 0.2	2.3 ± 0.8	58.9 ± 4.0

**Table 4:** Kinetics parameters extracted from the non-linear least-square fitting of the measured drug-release profiles of the ILDF particles using Equation (5).

Parameters	Description	Unit	Results
$k_{b}$	Burst constant	hr^−1^	1.599
$\theta_{b}$	Burst release fraction	-	0.00186
$D_{e}$	Diffusion Coefficient	cm^2^/s	2.341 × 10^−11^
$r_{p}$	Radius of particle	cm	0.00025
$\theta_{d}$	Diffusion release fraction	-	0.9981
$R^{2}$	Coefficient of determination	-	0.9905

## Data Availability

Not applicable.
